# The Insect Microbiome Modulates Vector Competence for Arboviruses

**DOI:** 10.3390/v6114294

**Published:** 2014-11-11

**Authors:** Natapong Jupatanakul, Shuzhen Sim, George Dimopoulos

**Affiliations:** 1 W. Harry Feinstone Department of Molecular Microbiology and Immunology, Bloomberg School of Public Health, Johns Hopkins University, 615 N. Wolfe Street, Baltimore, MD 21205, USA; E-Mail: njupata1@jhu.edu; 2 Genome Institute of Singapore, 60 Biopolis Street, #02-01 Genome, Singapore 138672, Singapore; E-Mail: shuzhens@gis.a-star.edu.sg

**Keywords:** mosquito, *Aedes*, *Culex*, arbovirus, innate immunity, microbiota

## Abstract

Diseases caused by arthropod-borne viruses (arboviruses), such as Dengue, West Nile, and Chikungunya, constitute a major global health burden and are increasing in incidence and geographic range. The natural microbiota of insect vectors influences various aspects of host biology, such as nutrition, reproduction, metabolism, and immunity, and recent studies have highlighted the ability of insect-associated bacteria to reduce vector competence for arboviruses and other pathogens. This reduction can occur through mechanisms, such as immune response activation, resource competition, or the production of anti-viral molecules. Studying the interactions between insect vectors and their microbiota is an important step toward developing alternative strategies for arbovirus transmission control.

## 1. Introduction

Over 130 arthropod-borne viruses (arboviruses) in the families *Togaviridae*, *Flaviviridae*, *Bunyaviridae*, *Reoviridae*, and *Orthomyxoviridae* can cause disease in humans [1]. Among these viruses, Dengue virus (DENV), West Nile virus (WNV), and Chikungunya virus (CHIKV) have become major global public health concerns, with increasing incidence in recent decades as a result of the expansion of the vectors’ geographic range, global transport, unplanned urbanization, and climate change [1,2,3,4,5,6].

Arboviruses are maintained in endemic areas by horizontal transmission between vertebrates and blood-feeding insect vectors. While arboviruses can cause serious pathology in humans, they have minimal impact on insect mortality. The insect immune system can control, but not clear, arbovirus infection; for this reason, infected insects can be vectors for life [7].

The replication cycle of arboviruses in insects has been extensively characterized; for example, DENV replication is well characterized in the *Aedes aegypti* mosquito [7]. After the mosquito ingests an infectious blood meal, the virus has to pass through various infection barriers [8]. It has to infect and replicate in the midgut epithelium (midgut infection barrier), then escape from the midgut to spread throughout the insect body and infect other tissues (midgut escape barrier). In order to transmit the disease, the virus then has to infect and replicate in the salivary glands and disseminate into mosquito saliva (salivary gland infection and escape barriers) [8]. The extrinsic incubation period (EIP), *i.e.*, the time from virus ingestion until its dissemination in mosquito saliva, where it can be transmitted to naïve humans, can vary depending on conditions such as mosquito strain, virus strain, and temperature, but it generally ranges from 7–14 days [7,9,10,11,12,13,14,15,16,17,18,19].

Insects constantly acquire microorganisms such as bacteria and fungi from their natural habitats and may also vertically acquire some species from their parents [20,21,22]. These diverse microbial communities affect multiple aspects of insect biology, such as nutrition, digestion, metabolism, development, and immunity, and, therefore, have great potential to alter vector competence for arboviruses [23,24,25,26,27].

Several studies of the microbiomes of the major mosquito vectors of arboviruses, *Ae. aegypti*, *Ae. albopictus*, and *Culex quinquefasciatus* have been performed along with analyses of anopheline microbiomes.

This review will summarize and discuss recent work on the interactions between the insect gut microbiota, insect host biology, and arboviruses, and how these studies may lead towards the development of alternative methods for arbovirus control.

## 2. Insect Microbiomes: Source, Dynamics, and Composition

Several studies have characterized the microbiomes of field mosquitoes using either culture-dependent or -independent methods [22,28,29,30,31,32]. The composition of the mosquito microbiome can vary depending on factors such as the species, sex, and life-stage of the mosquito, its geographical origin and feeding behavior, and the organ surveyed [22,28,29,30,31,32]. The relationship between these microbes and insects is complex and can range from pathogenesis to commensalism or mutualism [25,33].

### 2.1. The Mosquito Microbiome from Larvae to Adult

The mosquito life cycle consists of aquatic larval and pupal stages and a terrestrial adult stage. Because of these completely different habitats, the mosquito microbiome in different developmental stages can be distinct; this is particularly true for the gut. Mosquito gut contents are usually cleared when the insect undergoes metamorphosis and molting during the larvae-to-pupae and pupae-to-adult transitions [34], and the midgut microbiome in adult mosquitoes thus has to be repopulated. In the *Anopheles gambiae* mosquito, guts of aquatic stages have been found to be predominantly populated by *Cyanobacteria*, which serve as the larval diet [35,36,37]. On the contrary, adult *An. gambiae* guts are predominantly populated by Proteobacteria and Bacteroidetes picked up from the environment and ingested food after emergence [37]. There is also evidence that the gut microbiota is important for larval development. A recent study showed that when gut bacteria were depleted, mosquito larvae failed to molt and develop to the next stage [23]. Larval development could be restored by supplementing the breeding water with certain bacteria.

The adult mosquito gut microbiome has been the most extensively studied. While different mosquito species from the same geographical area share several core bacterial taxa, the composition of individual guts is highly variable [29]. The most common bacteria among different mosquito species from Kenya were Gammaproteobacteria (such as *Aeromonas*), Flavobacteria (such as *Chrysobacterium*), and Alphaproteobacteria (such as *Asaia*) [29]. A study of *Culex quinquefasciatus*, a mosquito vector for WNV, from India identified Proteobacteria (such as *Enterobacter*, *Pseudomonas*, *Pantoea*, and *Proteus*), Firmicutes (such as *Bacillus*), and Actinobacteria (such as *Acinetobacter*) as gut microbiota [38]. Studies in other mosquito species, such as *Ae. aegypti* and *Ae. albopictus*, insect vectors for DENV, CHIKV, and yellow fever virus (YFV), also identified Actinobacteria (such as *Streptomyces*, *Microbacterium*, and *Micrococcus*), Firmicutes (such as *Bacillus*), and Proteobacteria (such as *Asaia*, *Chromobacterium*, *Enterobacter*, *Pantoea*, *Pseudomonas*, and *Serratia*) [22,24,31,32,34,39,40].

The influence of the mosquito’s diet is reflected in the differences between male and female *Aedes* microbiomes [22,31]. Male mosquitoes acquire soil- and water-associated Actinobacteria through nectar feeding [22]. In female mosquitoes, however, bacteria in the phylum Proteobacteria, especially the family *Enterobacteriaceae*, which can tolerate redox stress from blood-meal digestion, are the main components of the midgut microbiome [22,37].

Although many studies have treated insects as holobionts, a few have tried to characterize the microbiota associated with individual organs, such as salivary glands, reproductive organs, and hemocoel [20,41,42,43,44]. Some microbes identified in non-gut tissues include intracellular bacteria such as *Wolbachia* (reproductive organs, salivary glands, head, muscle, and Malpighian tubules) and *Spiroplasma* (hemolymph, hemocytes, thoracic flight muscle, and nerve cells) [20,40,42]. These studies provide additional insight into how the microbiome can influence mosquito biology and vector competence. For example, *Wolbachia* in the salivary glands provides *Ae. albopictus* with resistance to DENV infection [45], and bacteria residing in reproductive organs have the potential for vertical transmission and will facilitate the administration of these microorganisms in the field [46,47,48]. *Spiroplasma,* a maternally-inherited endosymbiont extensively studied in *Drosophila*, has been found to cause pathology, influence insect reproduction, and alter the susceptibility of *Drosophila* to certain pathogens [49,50,51]. Pathogenicity of *Spiroplasma* in mosquitoes has also been documented [42,52,53,54,55]; however, the role of *Spiroplasma* in mosquito vector competence for arboviruses is as yet poorly studied.

### 2.2. Wolbachia and Cytoplasmic Incompatibility (CI)

Bacteria of the genus *Wolbachia* are maternally inherited, obligate intracellular symbionts that have been estimated to infect 66% of insects [56]. Several arbovirus vectors such as *Culex quinquefasciatus* and *Ae. albopictus* are naturally infected with *Wolbachia*, but not *Ae. aegypti* [43,57,58,59]. Recent research, however, has shown that stable transinfection of *Wolbachia* from *Drosophila* and *Ae. albopictus* into *Ae. aegypti* is possible [41,60,61] and, in fact, has great potential as an arboviral control strategy (described below in Section 3).

*Wolbachia* spreads quickly through populations because of its ability to alter insect reproduction through mechanisms such as feminization, parthenogenesis, and cytoplasmic incompatibility (CI), which increase the reproductive success of infected insects [62]. In mosquitoes, CI ensures that offspring will be infected by *Wolbachia* because uninfected eggs fertilized with sperm from infected males will not survive [63,64]. This phenomenon, which is maintained in stably trans-infected *Ae. aegypti*, is useful for the dissemination of *Wolbachia* in field mosquito populations [47].

### 2.3. The Insect Eukaryotic Microbiome

In addition to bacterial microbiota, studies have also isolated eukaryotic microorganisms such as fungi and yeast using culture-dependent methods. An early study identified 18 non-pathogenic yeast isolates in the genera *Candida*, *Yarrowia*, *Rhodotorula*, and *Cryptococcus* from larval and adult stages of *Aedes*, *Culex*, and *Anopheles* mosquitoes [65]. A later study isolated *Candida* and *Pichia* yeast from *Ae. aegypti* midguts [66]. *Wickerhamomyces anomalus* yeast has also been found in the midgut and reproductive organs of various mosquito species, suggesting a complex eukaryotic microbiome in various tissues [67,68]. These findings are not limited to mosquitoes. For example, 39 fungi were isolated from the cuticle and midgut of five sandfly species, suggesting that eukaryotic microbiota might be common among insects [69].

The eukaryotic microbiota has been much less well-studied than the bacterial microbiota, especially with metagenomic sequencing methods, and further work is required in order for us to fully understand its impact on insect biology and arbovirus transmission.

Paratransgenesis, which involves the genetic modification of insect microbiota to inhibit human pathogens, is considered a promising novel disease control approach [70,71]. The eukaryotic microbiome, especially yeast, have a high potential for paratransgenesis due to their safety, large scale production systems, and available genetic manipulation tools [72,73,74]. Yeasts can be genetically modified to inhibit arboviruses through secretion of antiviral anti-microbial peptides (AMP), such as a cecropin-like peptide possessing anti-DENV and anti-CHIKV activity [75]. Paratransgenesis can also be applied to entomopathogenic fungi, thus maximizing disease control potential through a combination of vector killing and reduction of vector competence. This approach has been employed with the fungus *Metarhizium anisopliae*, by engineering it to express the SM1 peptide, which inhibits *Plasmodium* development in *Anopheles* mosquitoes [76].

## 3. Microbiota-Driven Mechanisms Affecting Vector Competence

Insect microbiomes have long been co-evolving with their hosts. Early studies of insect symbionts suggested beneficial roles in nutrition; for example, the gut microbiota of termites greatly facilitate cellulose digestion [77,78]. Recent studies in medically important insect vectors also indicate the importance of microbiota for nutrient digestion, metabolism, egg production, development, and immune responses [23,25,26,27,79,80,81,82]. In other cases, endosymbionts, such as *Wolbachia* and *Spiroplasma*, may require nutrients from the host for efficient replication [83,84]. These interactions have great potential to influence vector competence for pathogens, since arboviruses require host factors and cellular machinery for their replication and are also controlled by insect immune responses. In addition to these indirect effects, microbiota may also directly interact with arboviruses, since some bacteria species are known to secrete anti-viral compounds [85,86,87,88]. A novel *Chromobacterium sp.* (*Csp_P*) species isolated from field-caught *Ae. aegypti* can reduce mosquito susceptibility to DENV infection when introduced to the mosquito midgut tissue [89]. Certain microbiota can also increase vector competence for arbovirus infection [90,91].

### 3.1. Immune System Modulation

The insect immune system relies mainly on innate immune responses, which recognize pathogen associated molecular patterns (PAMPs) through pattern recognition receptors (PRRs). Pathogen recognition activates immune signaling pathways such as the Toll pathway, the immune deficiency (IMD) pathway, and the Janus kinase/signal transducers and activators of transcription (JAK-STAT) pathway [92,93,94]. Activation of these immune signaling pathways triggers immune defense mechanisms, such as melanization, encapsulation, phagocytosis, apoptosis, and production of AMPs [95,96,97,98,99,100,101,102].

Each of these immune signaling pathways can be activated by a wide spectrum of microorganisms and viruses. The Toll pathway is activated in response to Gram-positive bacteria, fungi, and DENV [16,103,104,105,106,107,108,109]. The IMD pathway controls immune responses to bacteria, DENV, and the human *Plasmodium* parasite *P. falciparum* [16,75,110,111,112,113]. The JAK-STAT pathway is a cytokine-induced signaling pathway that plays important roles in insect anti-viral (DENV and WNV) immunity as well as immune responses to bacteria, fungi, and *Plasmodium* parasites [16,114,115,116,117,118,119]. Given the overlapping and broad-spectrum nature of immune signaling cascades, microbiota can activate insect immune responses and indirectly affect insect vector competence for arboviruses [39,109,119].

The role of the microbiome on mosquito immunity and vector competence was first characterized in *Anopheles* mosquitoes and *Plasmodium* parasites [120]. Transcriptomic comparison between septic and aseptic *An. gambiae* using microarrays identified a number of immune-related genes up-regulated in the presence of midgut microbiota, which subsequently resulted in lower susceptibility to *Plasmodium* infection in septic mosquitoes. This study provided a fundamental basis for subsequent studies concerning the effect of the mosquito microbiome on arbovirus infection.

The effect of the microbiome on insect vector competence for arboviruses has been studied in DENV and *Ae. aegypti* [39,109]. Removal of mosquito gut microbiota by treatment with antibiotics results in higher midgut DENV titers [109], and gene expression analysis has revealed that aseptic *Ae. aegypti* have lower levels of AMP gene expression (attacin, cecropin, defensin, and gambicin), suggesting a lower level of immune activation [109]. DENV infection of *Ae. aegypti* salivary glands induces the Toll and IMD pathways and results in the expression of a putative cecropin-like peptide with antibacterial, anti-DENV, and anti-CHIKV activity [75]. Subsequently, the bacterium *Proteus* sp. (*Prsp_P*), derived from the gut of field mosquitoes, has been shown to up-regulate AMP gene expression and confer increased resistance to DENV infection of the mosquito gut [39]. These results emphasize the overlap between antibacterial and antiviral insect immune responses.

*Wolbachia* contributes to *Drosophila’s* resistance to virus infection, and trans-infection of *Wolbachia* from *Drosophila* to *Ae. aegypti* also increases the mosquitoes’ resistance to DENV, CHIKV, YFV, and *Plasmodium* infection [57,61,121,122,123]. Introducing *Wolbachia* into a new insect host can elicit immune responses, as shown in the trans-infection of *w*Mel and *w*MelPop from *Drosophila* to *Ae. aegypti* [124]. It has, however, also been shown that *Wolbachia* provides protection against DENV infection in *Drosophila* without activating *Drosophila*’s immune response [124], suggesting that *Wolbachia* provides protection against arbovirus infection through both immunity-dependent and -independent mechanisms, depending on the combination of *Wolbachia* strain and insect host. *Wolbachia* strain *w*AlbB from *Ae. albopictus* has also been trans-infected into *Ae. aegypti* and shown to contribute to DENV resistance [41,125,126]. Gene expression analysis of *Ae. aegypti* infected with *w*AlbB has revealed *w*AlbB-induced production of reactive oxygen species (ROS), which in turn induce the activation of the Toll pathway [126].

In addition to immune signaling, RNA interference (RNAi) is another major insect anti-viral mechanism. In the canonical exogenous small interfering (siRNA) pathway, viral genomes are recognized and degraded based on sequence complementarity, through the action of Dicer2 (Dcr2) and the RNA-induced silencing complex (RISC) [127,128,129]. The components of the exogenous siRNA pathway are constitutively expressed in the cytoplasm, and there is to date no evidence that this mechanism can be activated by microorganisms other than viruses. However, insects also rely on other small RNA pathways, such as the Piwi-interacting RNA (piRNA) [130] and microRNA (miRNA) pathways [131,132], to restrict arbovirus infection. Recent studies have shown that *Wolbachia* *w*MelPop-CLA can alter the mosquito's miRNA profile [133], and can also alter mosquito gene expression through the induction of host microRNAs (miRNAs) [134,135]. The induction of miRNA aae-miR-12 promotes the growth of *Wolbachia* through a down-regulation of DNA replication licensing factor (MCM6) and the monocarboxylate transporter (MCT1) genes; the increased *Wolbachia* growth then reduces vector competence for DENV in a density dependent manner [134]. Aae-miR-2940, another miRNA induced by *Wolbachia*, suppresses *Ae. aegypti* DNA methyltransferase (AaDnmt2) gene expression. The down-regulation of AaDnmt2 again promotes *Wolbachia* replication but reduces DENV titers in mosquito cells [135].

The impact of microbiota on immune activity and vector competence in insects has also been documented in insects other than arbovirus vectors. The tsetse fly symbiont, *Wigglesworthia glossinidia*, activates the IMD pathway and inhibits trypanosome parasite infection [136]. Studies of *W. glossinidia* in tsetse suggest the importance of the microbiota in the larval stages for immune maturation in adult insects. Wild-type flies that lack *W. glossinidia* during larval development appear to have compromised immune responses such as AMP expression, prophenol-oxidase activity, melanization, and increased hemocyte number [81,137]. These results emphasize the importance of certain microbiota in particular developmental stages for maturation of the insect immune system in adults; however, this phenomenon is yet to be studied in arbovirus vectors.

In some cases, the insect microbiota does not confer resistance to arbovirus infection but instead increases the insects’ susceptibility to arbovirus infection. For example, trans-infection of *w*AlbB *Wolbachia* to *Culex tarsalis* increases the susceptibility of the mosquitoes to WNV infection [90]. Gene expression analysis has revealed down-regulation of Rel1, a transcription factor responsible for activating Toll pathway-dependent effectors, suggesting that *Wolbachia* can suppress insect immune responses [90]. The presence of *Serratia odorifera* in the *Ae. aegypti* midgut increases the mosquitoes’ susceptibility to DENV infection, possibly through a suppression of immune responses via the binding of prohibitin [91]; however, this possibility has yet to be experimentally confirmed. Other than arboviruses, recent studies have shown that *Wolbachia* increases mosquito susceptibility to *Plasmodium* parasite infection [138,139]. One *Wolbachia* strain can result in either an increase or a decrease of *Plasmodium* infection, for example, *w*AlbB reduces *P. falciparum* infection but increases infection of *An. gambiae* with *P. berghei* [139,140]. Environmental factors such as temperature can also affect the outcome of *Plasmodium* infection when mosquitoes are infected with *Wolbachia* [141]*.* For example, *Wolbachia w*AlbB reduces *P. yoelii* infection at 28 °C, but increase parasite load at 20 °C. These observations emphasize that the relationship between insect vector, insect microbiota, and human pathogens is far more complex than anticipated, and environmental factor can influence these interaction.

### 3.2. Resource Competition

Certain combinations of *Wolbachia* strain-insect species do not result in the elicitation of insect immune responses, as reported in *D. simulans* and *Ae. albopictus* [142] or in infections of Drosophila with the *w*Au and *w*Mel strains [143], suggesting that immune activation is not the only mechanism affecting vector competence. Both arboviruses and insect microbiota, especially endosymbionts, such as *Wolbachia* and *Spiroplasma*, require nutrients and host factors for efficient replication. The anti‑viral effect of *Wolbachia* in an *Ae. albopictus* cell line has been shown to be density-dependent [144,145], suggesting that high densities of *Wolbachia* competing for limited resources can affect vector competence.

Lipids, for example, are required by both the microbiota and arboviruses. Several arboviruses such as DENV and WNV use receptor-mediated endocytosis for cell entry in both vertebrate and invertebrate hosts, a process that involves remodeling of lipid membranes [146,147,148,149,150]. After cell entry, viruses modify intracellular compartments of the host to facilitate protein processing and virus replication and assembly [151,152]. DENV influences expression of genes involved in lipid synthesis to alter the host’s lipid composition, lipid homeostasis, and intracellular membrane trafficking [153,154,155]. *Wolbachia* also uses lipids from host cells for replication and therefore competes with and inhibits DENV and CHIKV replication [61,156].

In the *Drosophila* and honeybee models, *Spiroplasma* replication requires lipid and vitamins from its insect host [83,84]. Its requirements in mosquitoes have not been studied, but if similar to those of *Drosophila* and honeybees, it is plausible that the bacteria may also be able to influence vector competence through *Wolbachia*-like resource competition mechanisms.

### 3.3. Secondary Metabolite Production

Actinomycetes, bacteria commonly found in mosquito gut, have long been known to secrete secondary metabolites with anti-bacterial, anti-fungal, and anti-viral activity [88,157]. Another bacterium commonly found in soil and water, *Chromobacterium violaceum*, has also been studied for its anti-viral activity [86,158]. A recently characterized *Chromobacterium* sp. (*Csp_P*) isolated from field mosquito guts, has shown a promising potential as vector-borne disease control tool. *Csp_P* blocks infection of *An. gambiae* and *Ae. aegypti* with *Plasmodium* and dengue virus, respectively, and exerts entomopathogenic activity against larval and adult stages, likely though the production of secondary metabolites [89]*.* Bacteria isolated from the *Ae. albopictus* midgut, such as *Pseudomonas rhodesiae*, *Enterobacter ludwigii*, and *Vagococcus salmoninarium*, have been shown to directly inhibit La Crosse virus independently of the mosquito, suggesting that these bacteria may produce anti-viral molecules [159].

These discoveries suggest that certain species of disease vector’s natural gut microbiome directly influences arbovirus infection through natural products. Isolation of these bacteria and anti-pathogen molecules may open an interesting avenue for the discovery and development of novel therapeutic drugs.

## 4. Field Applications of Insect Microbiota for Arbovirus Transmission Control

The concept of insect microbiota as an arbovirus control tool has great potential, but it also raises numerous practical and safety concerns. In addition to exploring and characterizing anti-viral mechanisms, studies to address the applicability of these microorganisms to the field should be pursued. For example, the microbial composition of field mosquito guts can be far more complex than in those of mosquitoes in laboratory settings, and this complexity may interfere with the proposed arbovirus transmission-blocking strategy. This complexity was addressed in a recent study of the effect of the mosquito microbiome on the ability of *Wolbachia* to establish itself in a new insect host [160]. Interactions between the microbiota and *Wolbachia* inhibited transmission of *Wolbachia* to the next generation, and also resulted in mosquito mortality.

To date, the most advanced field application of insect microbiota for controlling arbovirus transmission is the Eliminate Dengue program in Australia, which has released *Wolbachia*-infected *Ae. aegypti* to control DENV transmission [47,60,161]. In this case, *Wolbachia* successfully invaded the natural mosquito population, and a follow-up study has found that field-caught *Wolbachia*-infected *Ae. aegypti* still maintain their refractoriness to DENV [162]. This program has since been expanded to other countries, including China, Vietnam, Indonesia, Colombia, and Brazil [163].

For gut bacteria which, unlike *Wolbachia*, are unable to drive themselves into a population, achieving sustained delivery to mosquitoes in nature remains an important and understudied practical issue. Existing measures for mosquito population control such as oviposition traps, spraying of toxins or insect pathogens, and artificial nectar bait [164,165,166,167,168,169] can be adapted as dissemination strategies; however, continued release may be required to maintain these microbiota in the mosquito population.

Paratransgenic approaches, while not explored in the area of arbovirus control, have the potential to reduce arbovirus transmission in a number of ways. Microbiota can be engineered to (1) have enhanced entomopathogenic activity [170,171], (2) secrete anti-pathogen molecules (extensively studied for arthropod-borne parasites) [70,71]), or (3) secrete molecules that activate insect immune responses against the pathogen. Research to identify candidate genes and molecules that increase entomopathogenic activity, inhibit arboviruses, and activate insect immune responses is required to move the field forward.

Collectively, research that will pave the way for the use of insect-derived bacteria as an alternative arbovirus control strategy is still at an early stage. Extensive studies are required to ensure safety and effectiveness prior to a field release of insect microbiota as an arbovirus control strategy.

## 5. Conclusion

The global burden of arboviral diseases has been rapidly increasing in recent decades. Studies of insect-associated microbial species suggest that they can alter vector competence by modulating host immune responses, competing with arboviruses for resources, and secreting anti-viral factors. Understanding the tripartite relationships between the insect, its microbiome, and the arboviral pathogens it harbors will allow us to develop alternative strategies to reduce the burden of arboviral diseases.
